# Supplementary material for: The Persian plateau served as hub for Homo sapiens after the main out of Africa dispersal
Source: Nat Commun. 2024 Mar 25;15:1882. doi: 10.1038/s41467-024-46161-7 (PMC10963722; doi:10.1038/s41467-024-46161-7)
Supplement: Supplementary file 1 — Supplementary Information [file 41467_2024_46161_MOESM1_ESM.pdf]

The Persian Plateau served as Hub for *Homo sapiens* after the main Out of Africa dispersal

Leonardo Vallini[1,c], Carlo Zampieri[1], Mohamed Javad Shoaee[2], Eugenio Bortolini[3], Giulia Marciani[3,4], Serena Aneli[5], Telmo Pievani[1], Stefano Benazzi[3], Alberto Barausse[1,6], Massimo Mezzavilla[1], Michael D. Petraglia[7,8,9], Luca Pagani[1,10,c]

1 Department of Biology, University of Padova, Italy
2 Department of Archaeology, Max Planck Institute for Geoanthropology, Jena, Germany
3 Department of Cultural Heritage, University of Bologna, Italy
4 Research Unit Prehistory and Anthropology. Department of Physical Sciences, Earth and Environment. University of Siena. Italy
5 Department of Public Health Sciences and Pediatrics, University of Turin, Italy
6 Department of Industrial Engineering, University of Padova, Italy
7 Human Origins Program, Smithsonian Institution, Washington D.C. 20560, USA
8 School of Social Science, The University of Queensland, Brisbane, Queensland, Australia
9 Australian Research Centre for Human Evolution, Griffith University, Brisbane, Queensland, Australia
10 Institute of Genomics, University of Tartu, Estonia
c to whom correspondence should be addressed: leo.vallini.lv@gmail.com, luca.pagani@unipd.it

Supplementary Information

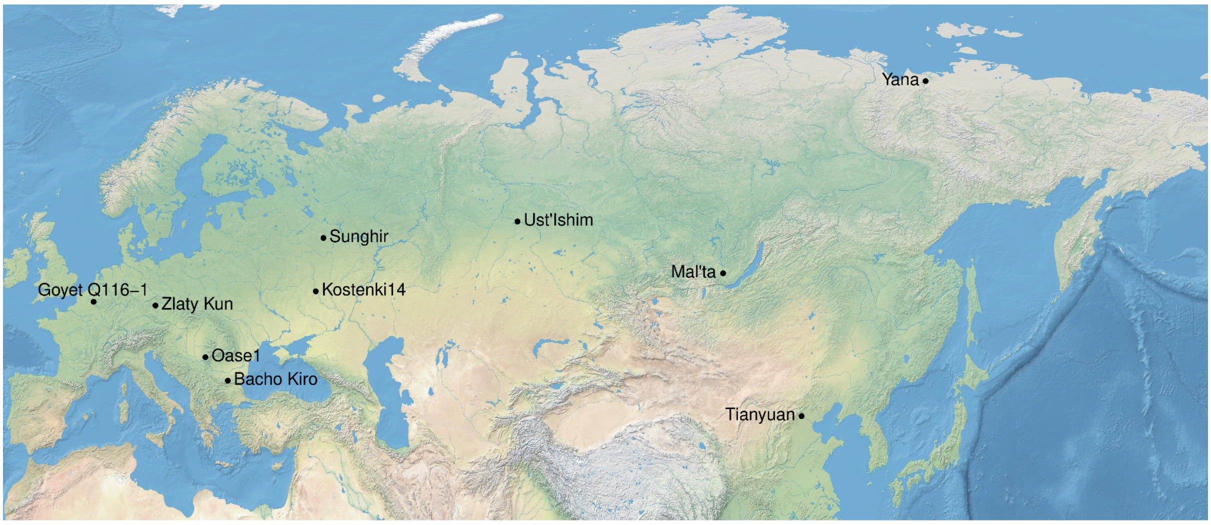

Supplementary Figure 1A: geographic position of the oldest paleolithic genomes analysed in the study

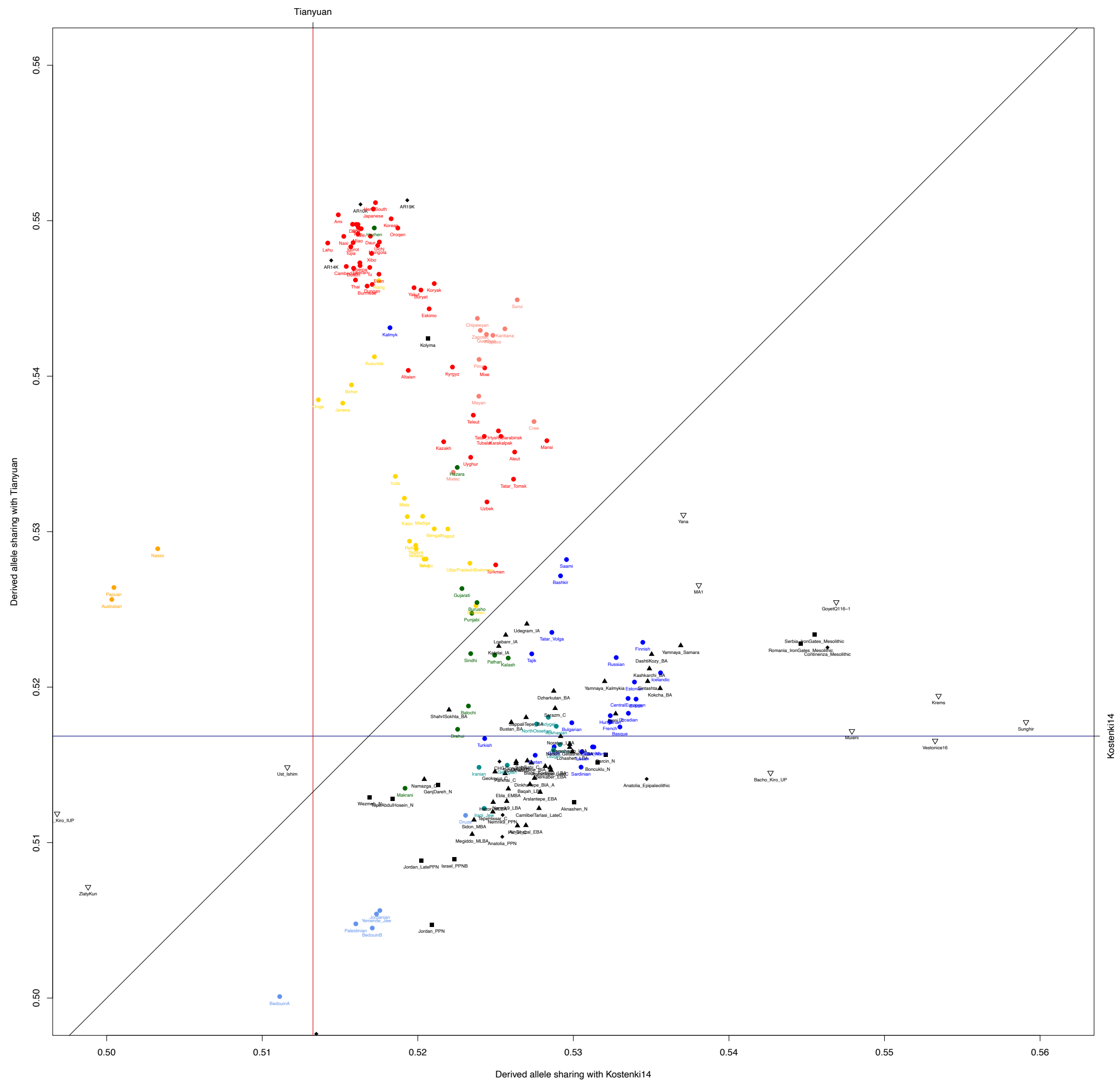

Supplementary Figure 1B: derived allele sharing of each individual/population with Kostenki14 and Tianyuan; Sub Saharan Africans are not shown to avoid shrinking the image too much. Source Data in Supplementary Data 3. East Asians in red, Oceanians in orange, Native Americans in pink, South Asians in yellow, Northern South Asians in green, West Eurasians in blue, Levantines in cornflowerblue, ancient samples in black.

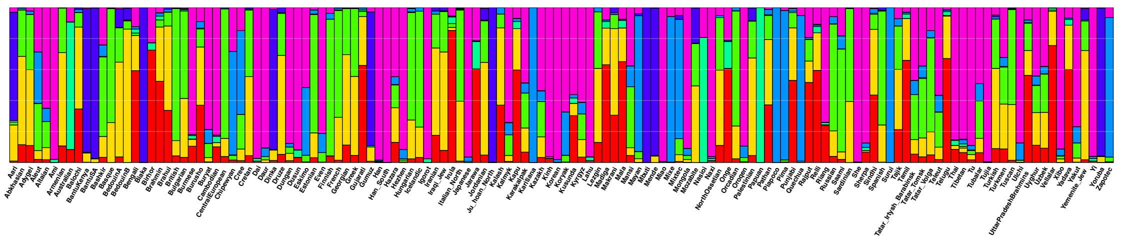

Supplementary Figure 2: Admixture with K=7 (lowers cv score for k ranging 2-10) of modern populations used in our analyses. Source Data in Supplementary Data 4.

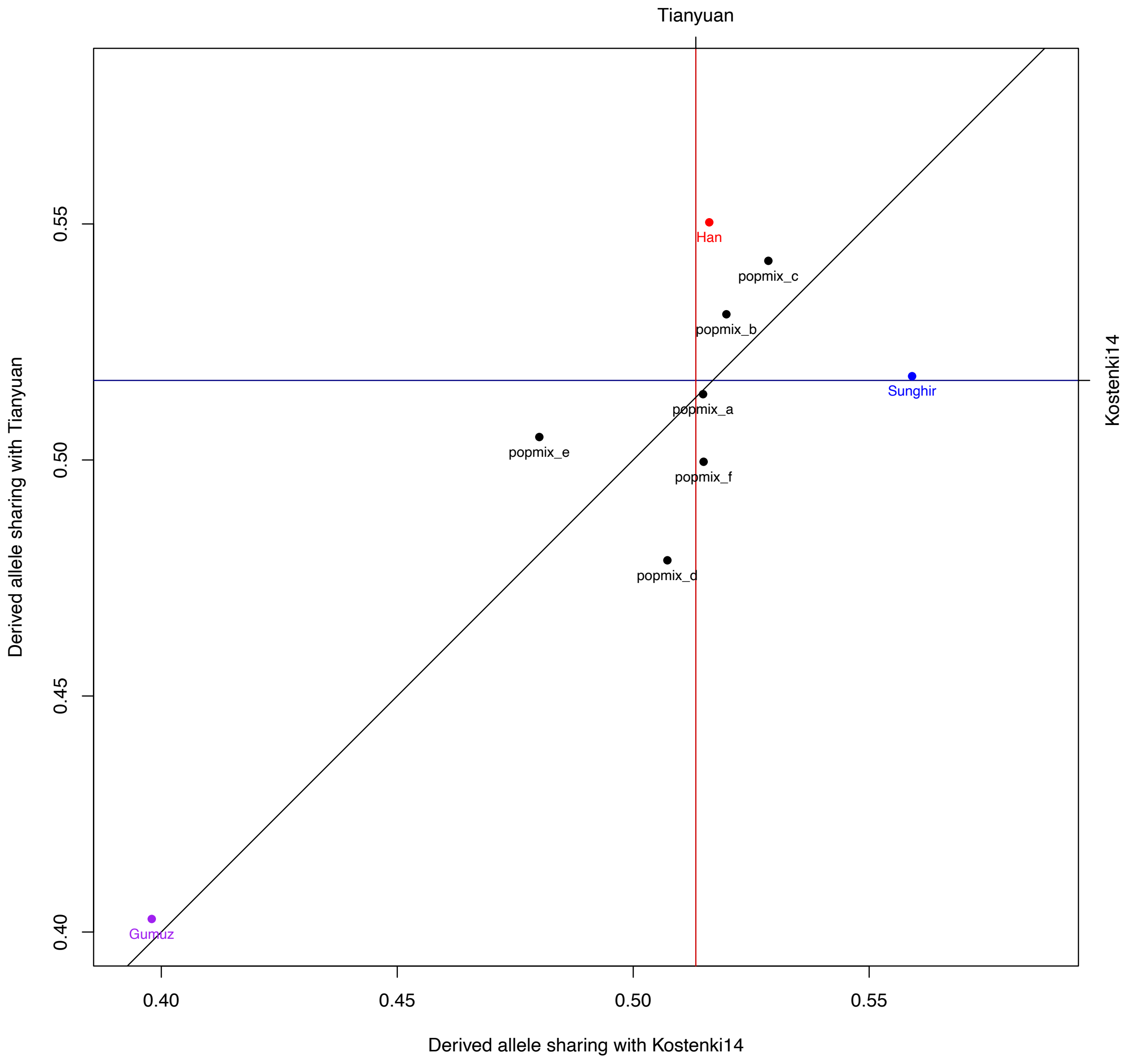

Supplementary Figure 3: DAS with Kostenki14 and Tianyuan of the artificially admixed populations and sources. Source Data in Supplementary Data 8.

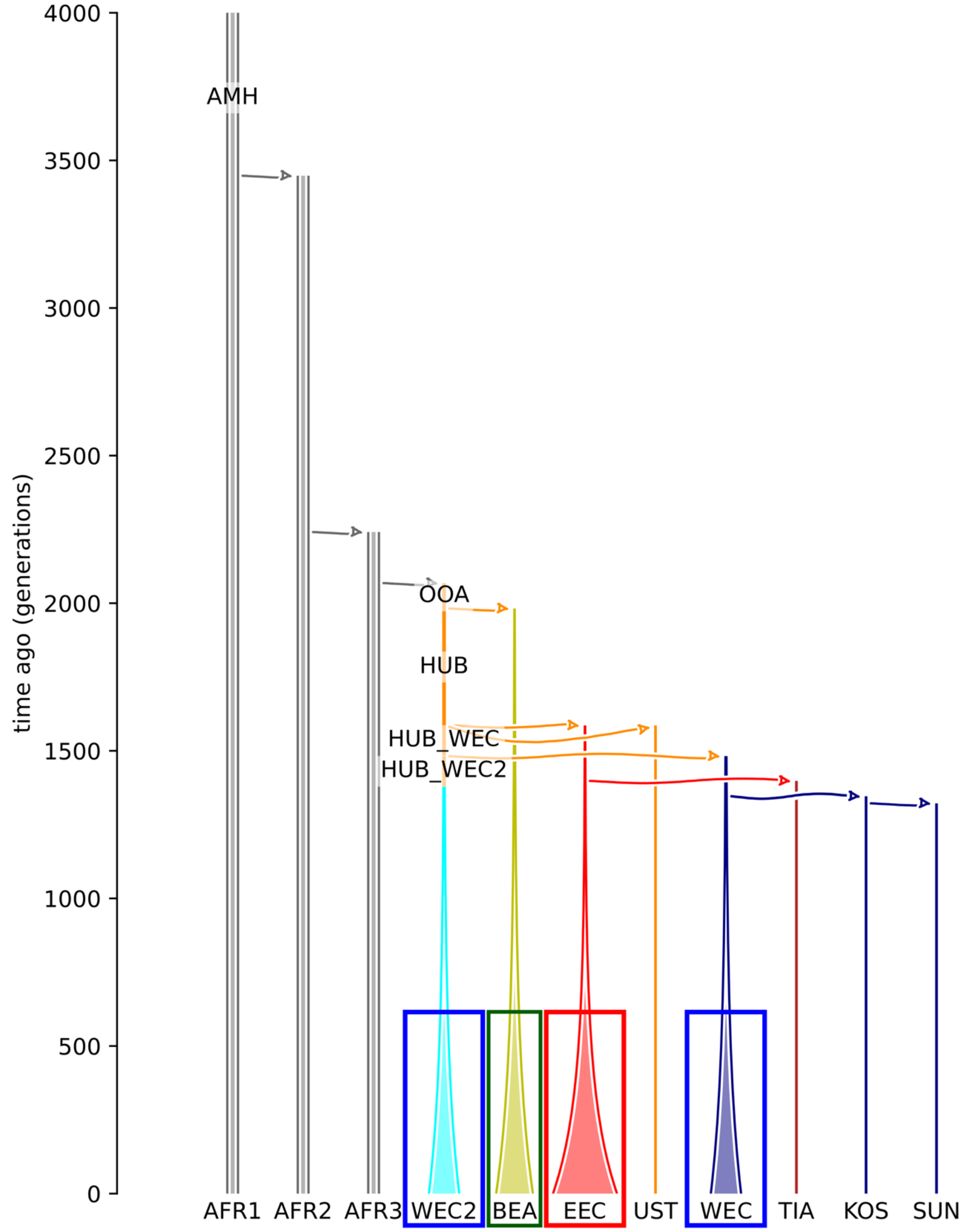

Supplementary Figure 4A: demographic model of the core(i.e. not showing admixed populations to avoid confusion) of our coalescent simulations, populations in boxes are the sources for all admixed populations. Details on admixture proportions of the admixed populations are given in Supplementary Data 9.

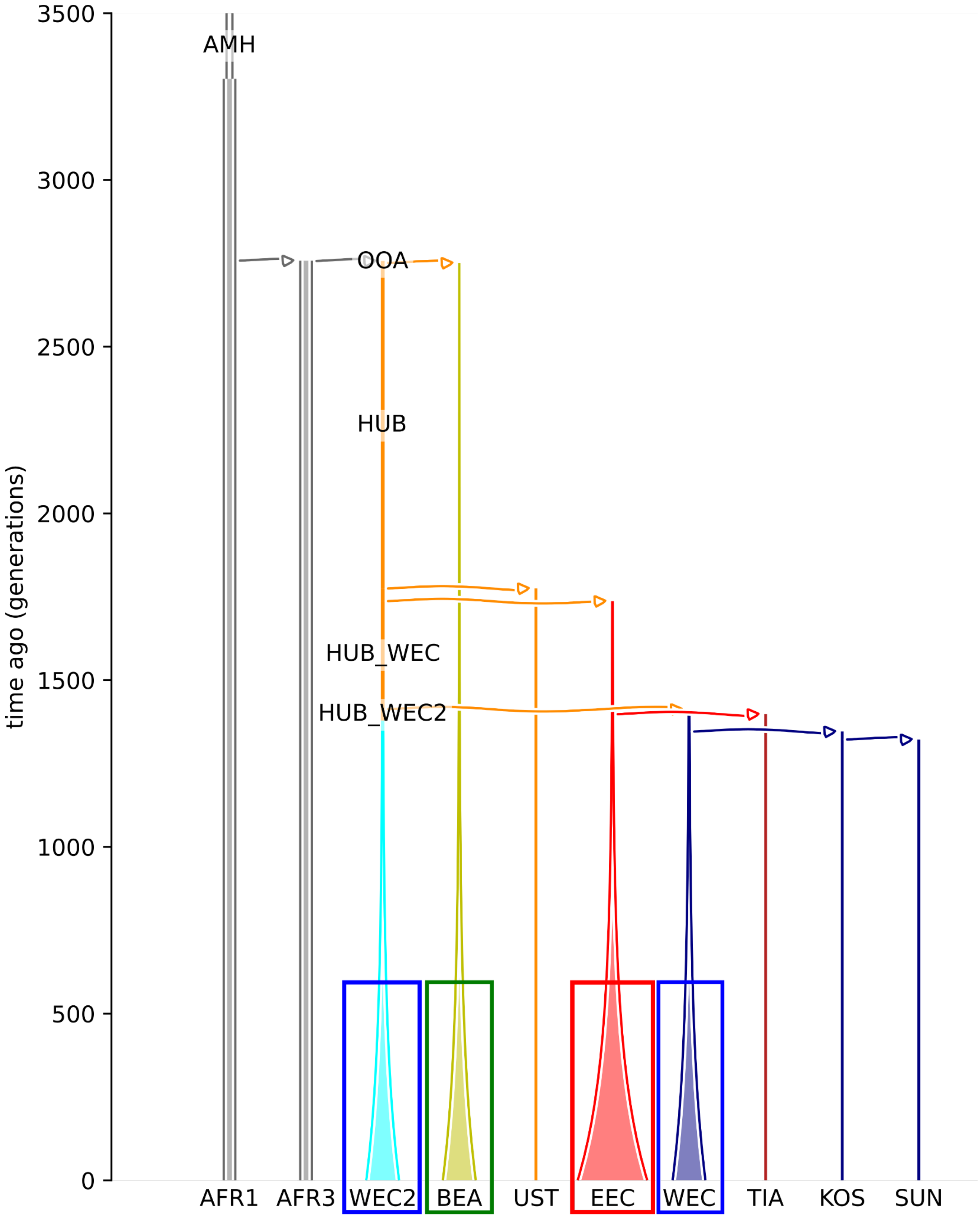

Supplementary Figure 4B: demographic model of the core(i.e. not showing admixed populations to avoid confusion) of our coalescent simulations, under the topology proposed by Kamm and colleagues. Populations in boxes are the sources for all admixed populations. Details on admixture proportions of the admixed populations are given in Supplementary Data 9.

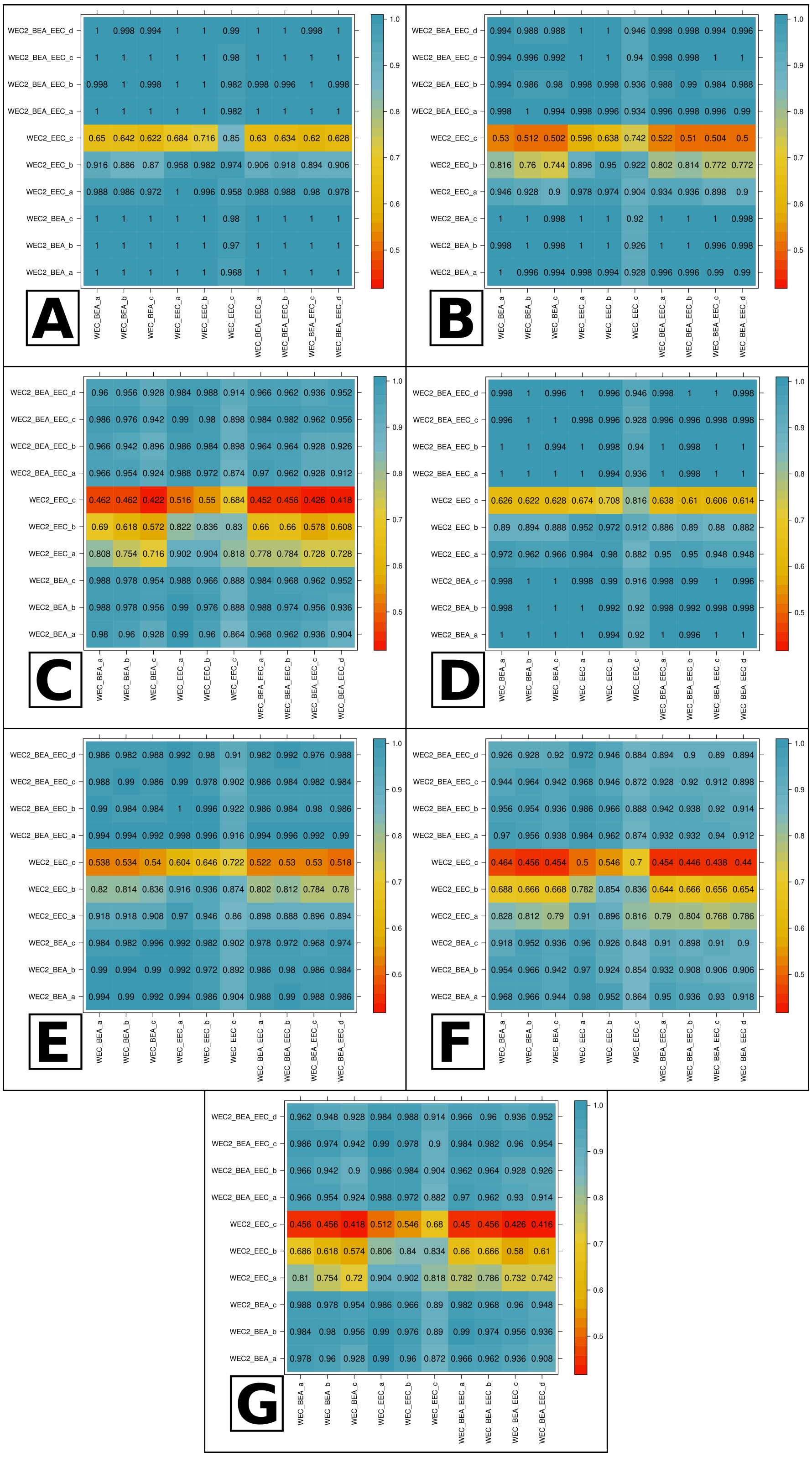

Supplementary Figure 5: matrices showing the proportion of times in which, for each pairwise comparison, we were able to retrieve the correct ranking of source populations when the most distant source split off after 3000 (panel A), 2000 (panel B) or 1000 (panel C) years from the Kostenki14 lineage. The results obtained using the topology put forward by Kamm and colleagues with the most distant source splitting off after 3000 (panel D), 2000 (panel E) or 1000 (panel F). Analysis of panel C repeated by keeping only SNPs with maf > 0.05 in simulated modern Eurasians to test the effect of ascertainment bias (panel G).

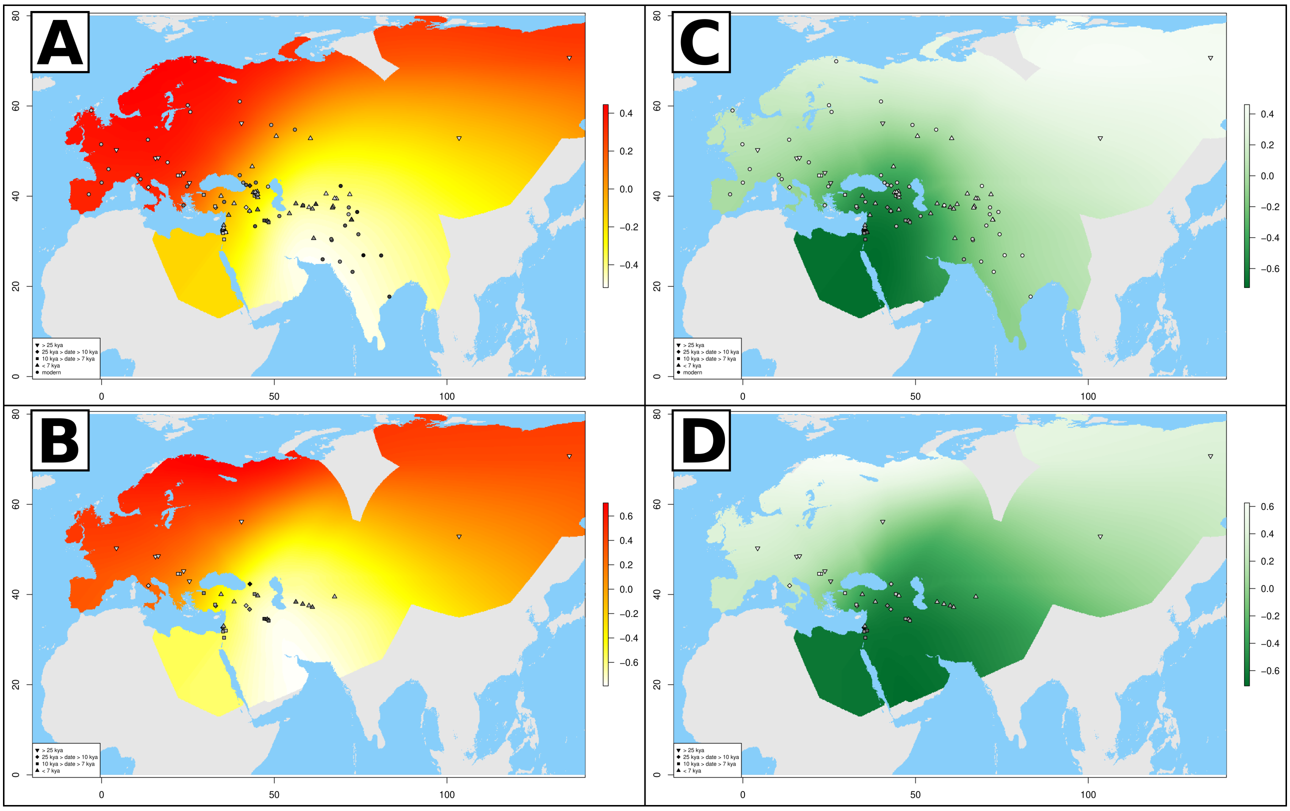

Supplementary Figure 6: Focal area analysis for the Hub (panel A, white to light yellow hues show the most likely Hub location from a genetic perspective) and for Basal Eurasians ancestry (panel C, dark hues show higher proportion of a Basal Eurasian component) when keeping all populations with more than 50% West Eurasian ancestry. Focal area analysis for the Hub (panel B) and for Basal Eurasians (panel D) when keeping only individuals older than five thousand years (panel C). Source Data in Supplementary Data 11.

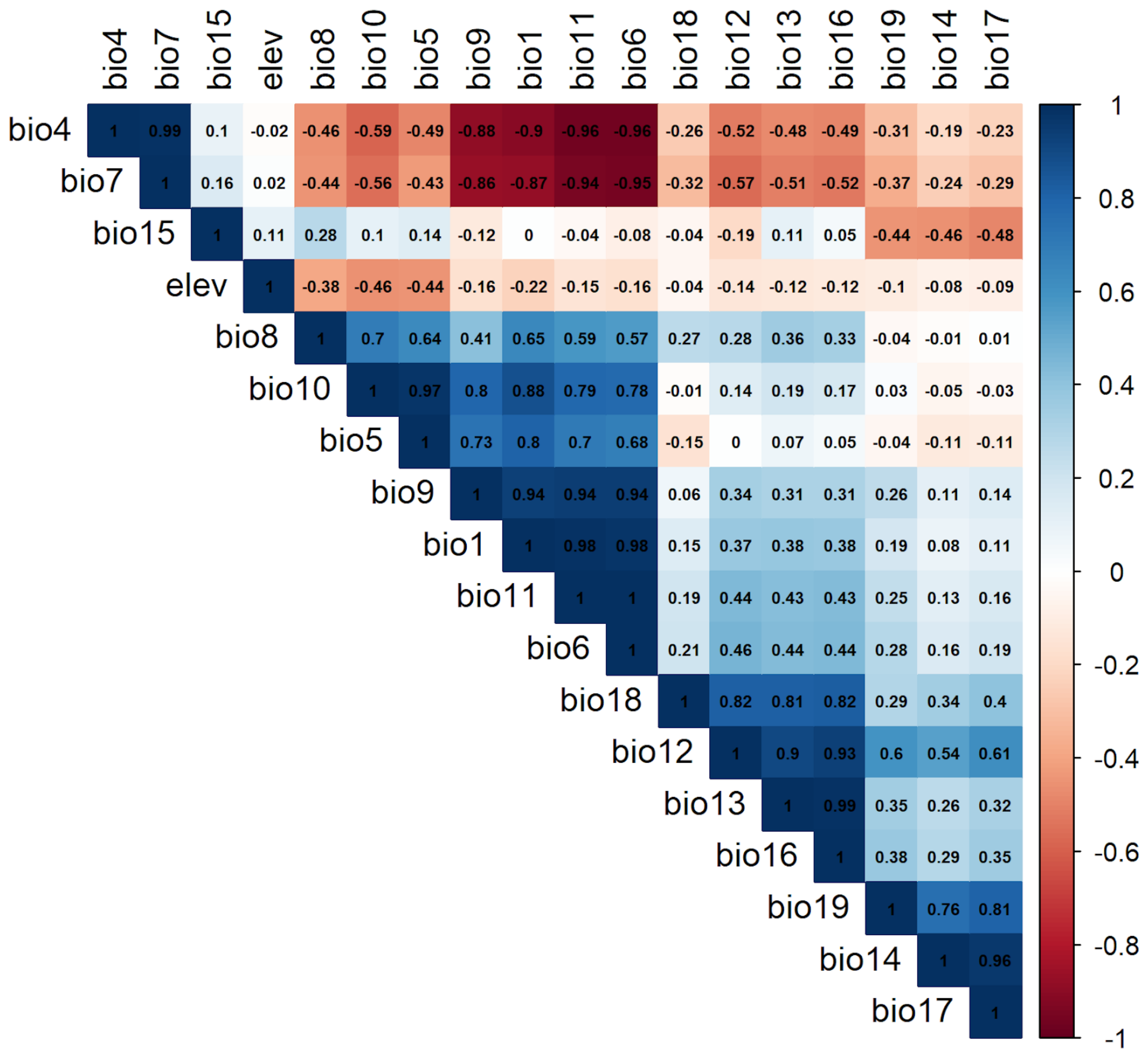

Supplementary Figure 7: Covariates extraction and selection process. Pairwise Pearson's correlation coefficients (r) between climatic and topographic variables computed on the whole studied area (883260 spatial points). Paleoclimatic variables abbreviations are reported in Supplementary Data 12

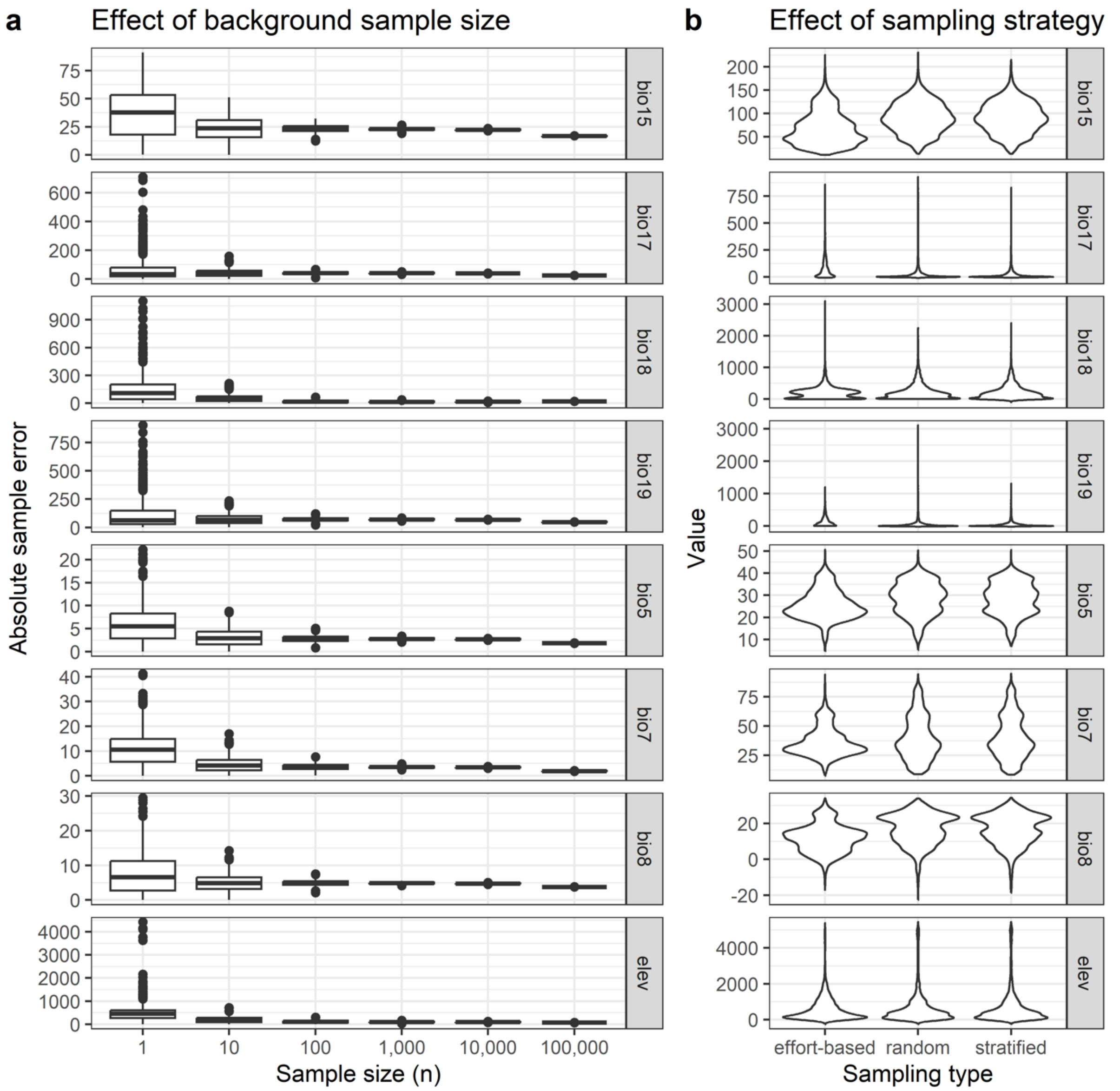

Supplementary Figure 8: Background points sampling. Effect of (a) sample size and (b) sampling strategy on the background points. Paleoclimatic variables abbreviations are reported in Supplementary Data 12.

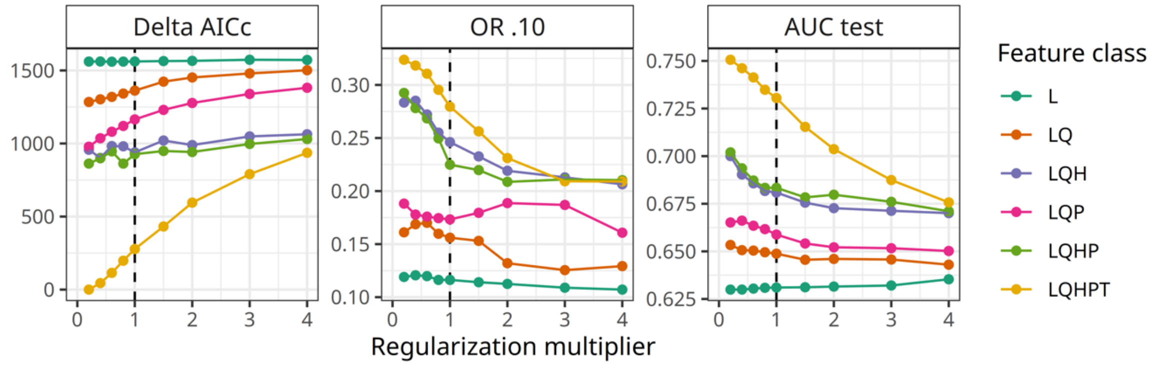

Supplementary Figure 9: Model configurations and selection. Candidate models performance measured with the corrected Akaike Information Criterion (AICc), the Omission Rate of the testing points at 10% training threshold, and the Area Under the receiver-operator-Curve (AUC). Colors represent the feature classes used, while on the x-axis it is reported the applied regularization multiplier (RM). Default model with default RM (RM = 1.0) is indicated by the vertical dashed line.

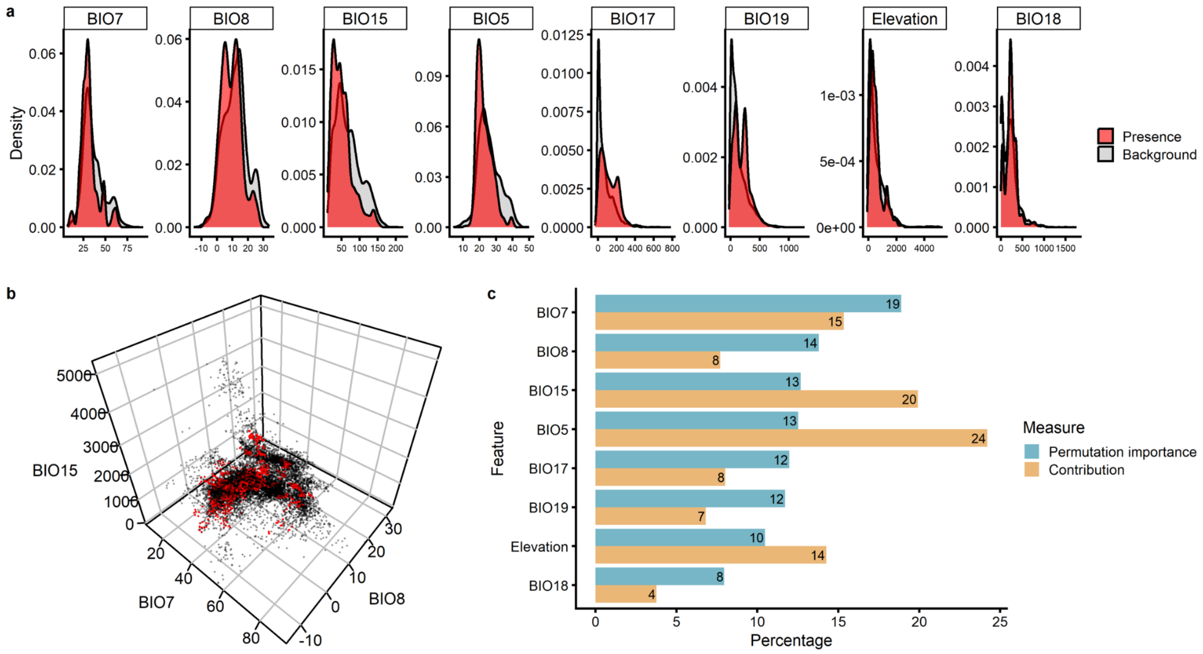

Supplementary Figure 10: (a) Density distribution of selected variables at presence points (red) and background points (grey). (b) Human niche (red) and background points (grey) described in a covariate space with the 3 most important predictors. (c) Covariates importance in terms of permutation importance (blue) and model contribution (yellow), both calculated by the MaxEnt routine implemented in R package ENMeval [1]. Paleoclimatic variables abbreviations are reported in Supplementary Data 12.

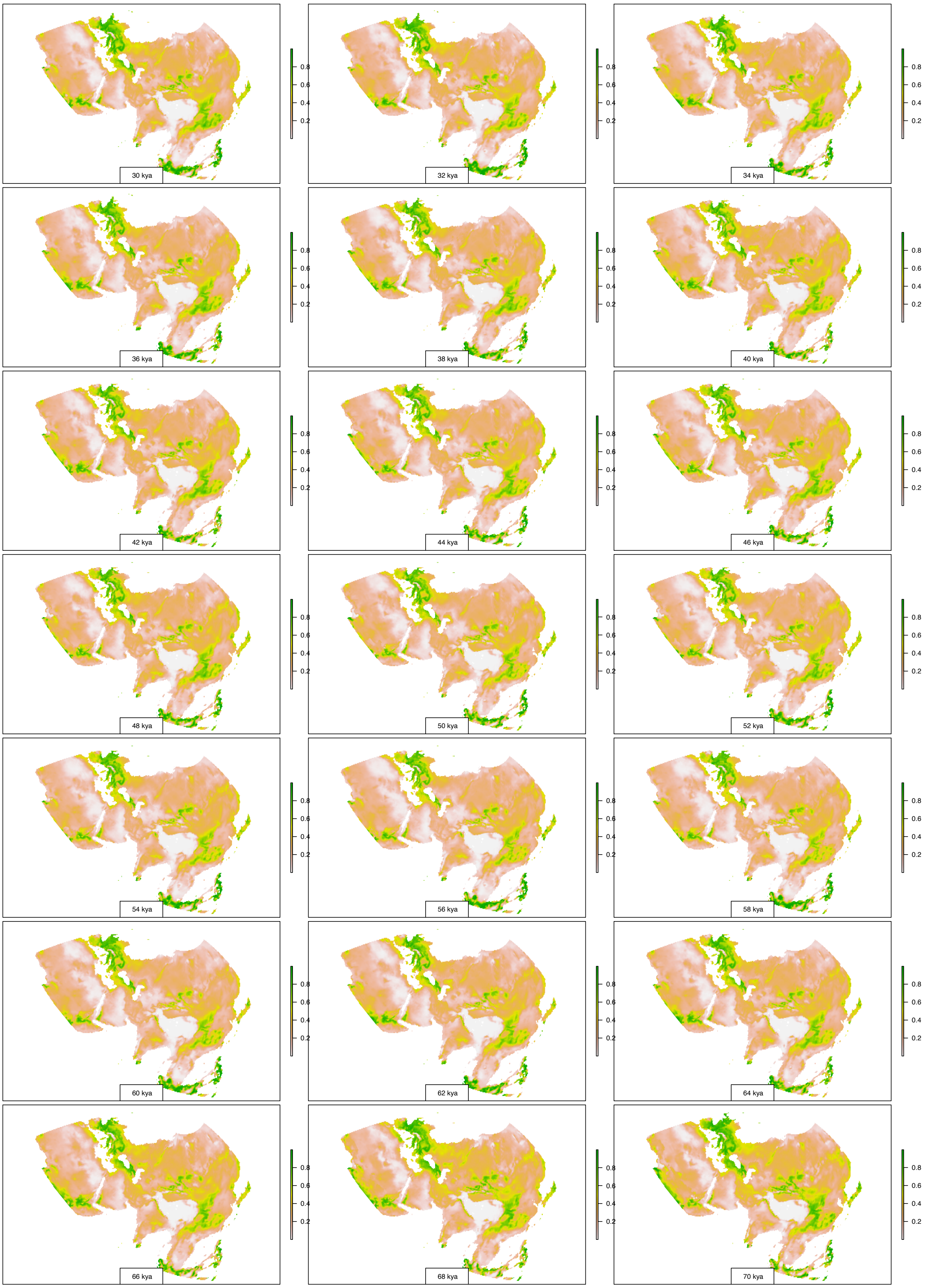

Supplementary Figure 11: Suitability for *Homo sapiens* occupation (1 = most suitable, 0 = least suitable) resulting from model predictions at different times during the period 70 kya to 30 kya (thousands of years ago).

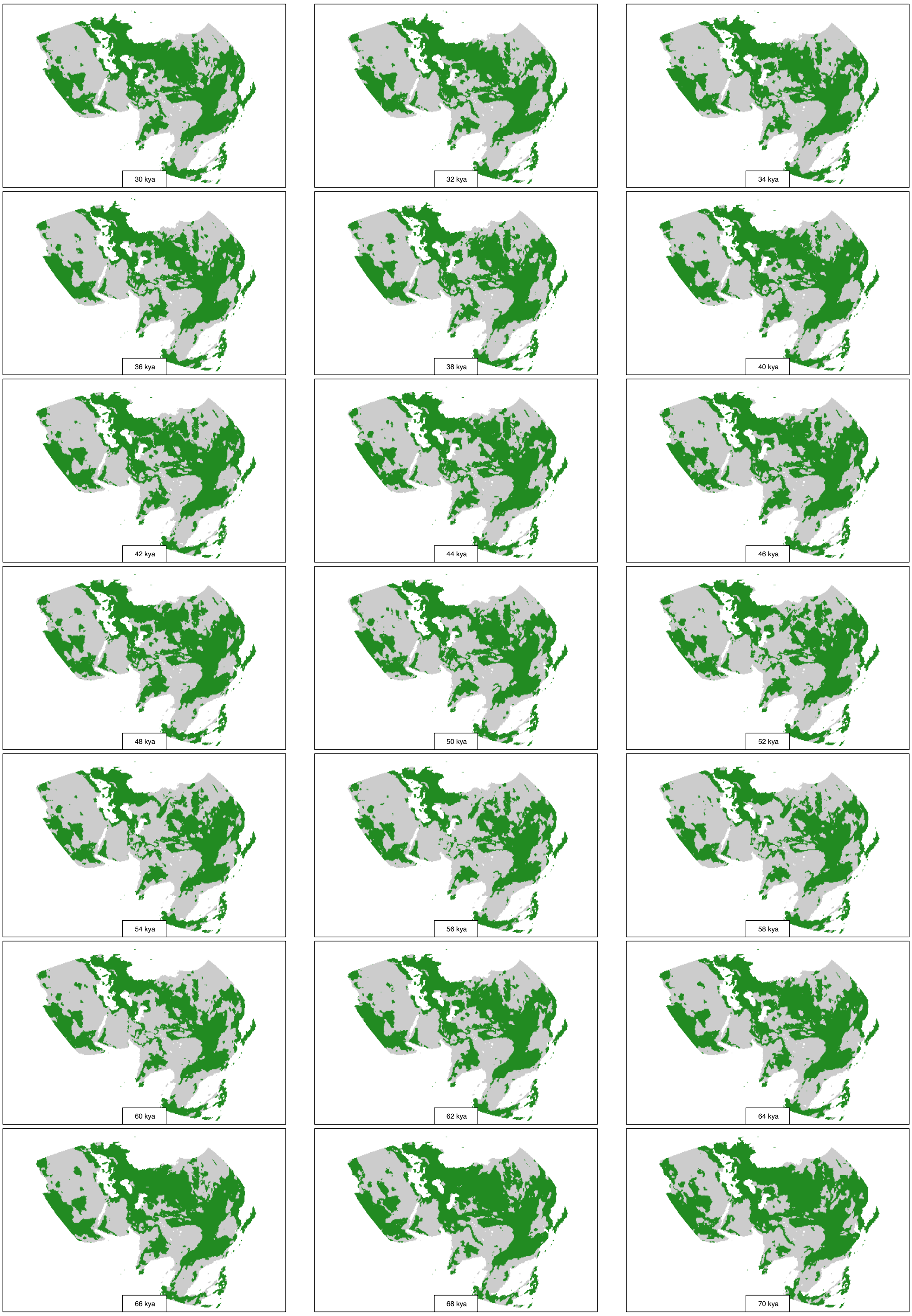

Supplementary Figure 12: Areas predicted as suitable (green) for *Homo sapiens* occupation during the period 70 kya to 30 kya (thousands of years ago), resulting from binarized probability surfaces (Supplementary Figure 11). Areas were considered suitable when suitability was higher than 0.238, which corresponds to the 5%-percentile of predicted values at observed sites [2,3].

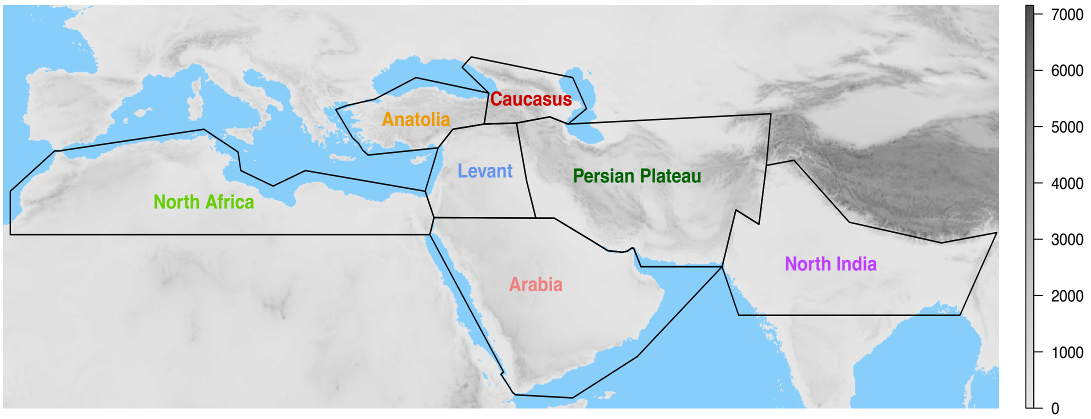

Supplementary Figure 13: boundaries of the geographic regions for which we computed the carrying capacity.

Supplementary Note 1

Skin pigmentation varies among different human populations and is thought to be under selection, due to differences in UV variation between latitudes. However, it is not clear whether similar skin phenotypes found at comparable latitudes are to be seen as examples of convergent evolution propelled by the same environmental stressor, or perhaps as the result of stabilizing selection acting on the dark skin phenotype that characterized the first Eurasian settlers on their journey out of Africa.We investigated the pattern of genetic differentiation in various populations on markers linked to genes and variants associated with skin pigmentation in ethnically diverse African populations as previously described by Crawford and colleagues [4]. We estimated the average Fst between Yoruba, taken as proxy for the allele frequency of the considered markers prior to the Out of Africa bottleneck, and all other populations of HGDP [5] and GenomeAsia100K [6] for a total of 930 markers linked to skin pigmentation. Then we estimated the average Genomic Fst between Yoruba and other target populations using a set of 10.000 randomly selected autosomal SNPs covered by both HGDP and GenomeAsia100K dataset, which we used as a proxy for genetic drift compared to our West African reference population. Finally, we normalized the Pigmentation Fst and Genomic Fst between 0 and 1, allowing a direct comparison between the two metrics. If the genomic Fst is similar to the pigmentation Fst it means that only genetic drift acted on the variation of these SNPs after the Out Of Africa, which is represented by the red line in Supplementary Figure 14. In our analyses West Eurasia populations (blue) show higher Fst for pigmentation markers than the genomic one, indicating increased drift or natural selection at these sites compared to Yoruba. On the contrary, Oceanian (red) and certain South (green) and South East (light blue) Asian populations, particularly Onge, Jarwa and Aeta showed a lower Fst for Pigmentation SNPs than the genomic Fst, indicating that these populations had a reduced genetic differentiation at pigmentation loci after they left Africa. Such a result may imply ongoing purifying or stabilizing selection directed towards the pigmentation phenotype, which is consistent with a scenario of an early dispersal of these groups from the Hub location (>45 kya, as part of the EEC expansion), which provided scope for the adaptive African pigmentation phenotype to remain under a similar selective pressure exerted by the UV radiation when these population moved to the low latitudes of the Eurasian and Oceanian continents.

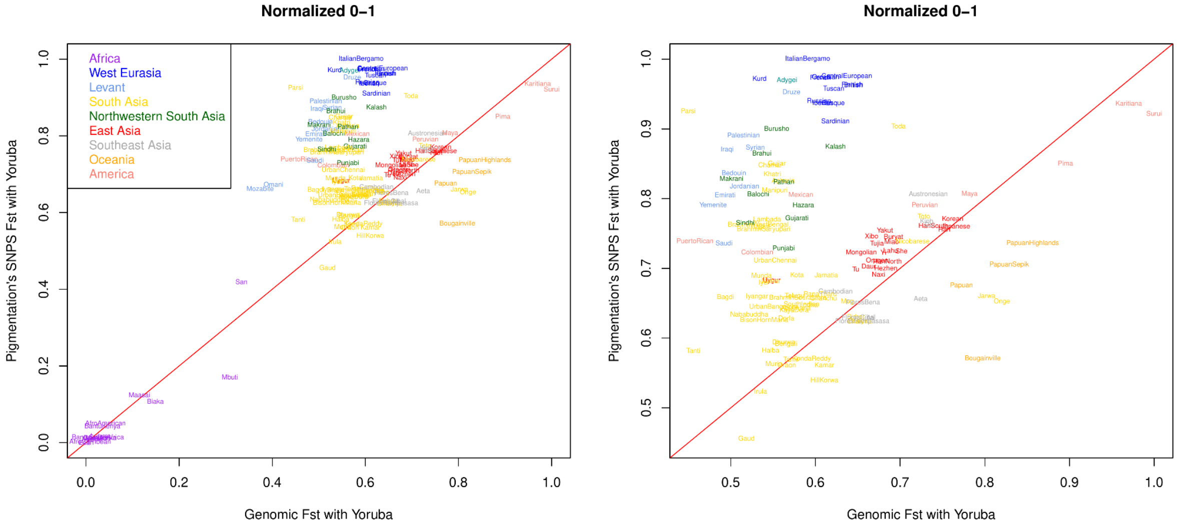

Supplementary Figure 14. Comparison of normalized Genomic and Pigmentation Fst between Yoruba and other populations from HGDP and GenomeAsia100Kin all populations (left panel) and only non-Africans (right panel). Source Data in Supplementary Data 15.

Supplementary References

1. Muscarella, R. *et al.* ENMeval: An R package for conducting spatially independent evaluations and estimating optimal model complexity forMaxentecological niche models. *Methods Ecol. Evol.* **5**, 1198–1205 (2014).
2. Banks, W. E. *et al.* An ecological niche shift for Neanderthal populations in Western Europe 70,000 years ago. *Sci. Rep.* **11**, 5346 (2021).
3. Rodríguez, J., Willmes, C., Sommer, C. & Mateos, A. Sustainable human population density in Western Europe between 560.000 and 360.000 years ago. *Sci. Rep.* **12**, 1–14 (2022).
4. Crawford, N. G. *et al.* Loci associated with skin pigmentation identified in African populations. *Science* **358**, (2017).
5. Bergström, A. *et al.* Insights into human genetic variation and population history from 929 diverse genomes. *Science* **367**, (2020).
6. GenomeAsia100K Consortium. The GenomeAsia 100K Project enables genetic discoveries across Asia. *Nature* **576**, 106–111 (2019).
