# Supplementary material for: The Persian plateau served as hub for Homo sapiens after the main out of Africa dispersal
Source: Nat Commun. 2024 Mar 25;15:1882. doi: 10.1038/s41467-024-46161-7 (PMC10963722; doi:10.1038/s41467-024-46161-7)
Supplement: Supplementary file 3 — Description of Additional Supplementary Files [file 41467_2024_46161_MOESM3_ESM.pdf]

Description of Additional Supplementary Materials

Supplementary Data 1: individuals used in this study

Supplementary Data 2: metadata on the populations used in our analyses

Supplementary Data 3: derived allele sharing with Kostenki14, Tianyuan and Ust'Ishim of each population analysed

Supplementary Data 4: unsupervised admixture results with K=7 (lowest cv score)

Supplementary Data 5: D-statistic results to assess whether ancient populations show a contribution from either East (Gumuz as a proxy) or West (Yoruba as a proxy) African populations.

Supplementary Data 6: individuals used as source for the artificially mixed populations

Supplementary Data 7: ancestry composition of the artificially mixed populations. The *_perc row for shows the percentage of the genome coming from each source population, while the *_chrs row indicates that those are the chromosomes from each source used in the mix

Supplementary Data 8: empirical (obs) and analytical (exp) derived allele sharing values of the artificially admixed and source populations.

Supplementary Data 9: source proportions in the admixed populations generated in our coalescent simulations, each population was simulated using both WEA and WEA2 as source

Supplementary Data 10: D-statistic results to assess the East Eurasian population (either Han or ASI) that contributed to each admixed population. When the statistic is significantly negative the row is highlighted in red, when it is significantly positive in yellow and when it does not significantly deviate from zero in grey

Supplementary Data 11: KB and proportion of BEA, WEC and EEC for all population analysed with pWEA > 0.5 (as in Supplementary Figure 6A and 6C). The list is further restricted to individuals with pWEC >0.75 for Figure 2, and to individuals older than 5 ky for Supplementary Figure 6B and 6D.

Supplementary Data 12: Variables and their abbreviations extracted from Beyer et al. 2020 (adapted from pastclim package, Leonardi et al. 2022).

Supplementary Data 13: Multicollinearity indices (R2) tolerance (TOL=1-R2) and Variance Inflation Factor (VIF) of the environmental variables for each selection step. In step 1 only variables with Pearson's |r|<0.9 were retained (see Supplementary Figure X1). In step 2 the variable with the highest VIF was iteratively excluded until all the remaining ones had VIF<5.

Supplementary Data 14: estimates of the carrying capacity of each region in each time interval using the regression equations for Hunters shown in Table 1 of Rodriguez et al. 2022

Supplementary Data 15: Genomic and pigmentation related SNPs Fst with Yoruba, normalised

Supplementary Code 1: python code to run the coalescent simulations under the demographic model shown in Supplementary Figure 4A

Supplementary Code 2: python code to run the coalescent simulations under the demographic model shown in Supplementary Figure 4B
