# Supplementary material for: The Persian plateau served as hub for Homo sapiens after the main out of Africa dispersal
Source: Nat Commun. 2024 Mar 25;15:1882. doi: 10.1038/s41467-024-46161-7 (PMC10963722; doi:10.1038/s41467-024-46161-7)
Supplement: Supplementary file 6 — Reporting Summary [file 41467_2024_46161_MOESM6_ESM.pdf]

nature portfolio

Corresponding author(s): Luca Pagani

Last updated by author(s): Jan 29, 2024

Reporting Summary

Nature Portfolio wishes to improve the reproducibility of the work that we publish. This form provides structure for consistency and transparency in reporting. For further information on Nature Portfolio policies, see our Editorial Policies and the Editorial Policy Checklist.

Statistics

For all statistical analyses, confirm that the following items are present in the figure legend, table legend, main text, or Methods section.

n/a	Confirmed	
☐	☒	The exact sample size (*n*) for each experimental group/condition, given as a discrete number and unit of measurement
☐	☒	A statement on whether measurements were taken from distinct samples or whether the same sample was measured repeatedly
☐	☒	The statistical test(s) used AND whether they are one- or two-sided<br>*Only common tests should be described solely by name; describe more complex techniques in the Methods section.*
☐	☒	A description of all covariates tested
☐	☒	A description of any assumptions or corrections, such as tests of normality and adjustment for multiple comparisons
☐	☒	A full description of the statistical parameters including central tendency (e.g. means) or other basic estimates (e.g. regression coefficient) AND variation (e.g. standard deviation) or associated estimates of uncertainty (e.g. confidence intervals)
☐	☒	For null hypothesis testing, the test statistic (e.g. *F*, *t*, *r*) with confidence intervals, effect sizes, degrees of freedom and *P* value noted<br>*Give P values as exact values whenever suitable.*
☒	☐	For Bayesian analysis, information on the choice of priors and Markov chain Monte Carlo settings
☒	☐	For hierarchical and complex designs, identification of the appropriate level for tests and full reporting of outcomes
☒	☐	Estimates of effect sizes (e.g. Cohen's *d*, Pearson's *r*), indicating how they were calculated

Our web collection on statistics for biologists contains articles on many of the points above.

Software and code

Policy information about availability of computer code

Data collection	No software was used for data collection
Data analysis	plink (https://www.cog-genomics.org/plink/)<br>admixtools (https://github.com/DReichLab/AdmixTools)<br>admixture (https://dalexander.github.io/admixture/)<br>msprime (https://tskit.dev/msprime/docs/stable/installation.html) - custom code for coalescent simulations is available as a supplementary note<br>R (https://cran.r-project.org/)<br>R packages: pastclim (https://cran.r-project.org/web/packages/pastclim), Hmisc (https://cran.r-project.org/web/packages/Hmisc), fuzzySim (https://cran.r-project.org/web/packages/fuzzySim), ks (https://cran.r-project.org/web/packages/ks), ENMeval (https://cran.r-project.org/web/packages/ENMeval), terra (https://cran.r-project.org/web/packages/terra), raster (https://cran.r-project.org/web/packages/raster), marmap (https://cran.r-project.org/web/packages/marmap), ncdf4 (https://cran.r-project.org/web/packages/ncdf4), oce (https://cran.r-project.org/web/packages/oce), ocedata (https://cran.r-project.org/web/packages/ocedata)

For manuscripts utilizing custom algorithms or software that are central to the research but not yet described in published literature, software must be made available to editors and reviewers. We strongly encourage code deposition in a community repository (e.g. GitHub). See the Nature Portfolio guidelines for submitting code & software for further information.

Data

Policy information about availability of data

All manuscripts must include a data availability statement. This statement should provide the following information, where applicable:

- Accession codes, unique identifiers, or web links for publicly available datasets
- A description of any restrictions on data availability
- For clinical datasets or third party data, please ensure that the statement adheres to our policy

No new data was produced for this study.
Genomic data for modern and ancient individuals is available from the Allen Ancient DNA Resource_(https://reich.hms.harvard.edu/allen-ancient-dna-resource-aadr-downloadable-genotypes-present-day-and-ancient-dna-data). Gumuz genomic data is available from the European Genome-Phenome Archive under accession number EGAS00001000238.
Alti-bathimetric geographic maps of the ETOPO1 dataset 108,109 and the inferred sea levels in the past from the "Global 1Ma Temperature, Sea Level, and Ice Volume Reconstructions" dataset 110 are available from the NOAA Paleoclimatology Program. Paleoclimatic Data 57 can be accessed using the R package Pastclim 58.
The data to plot Figure 1C is reported in Supplementary Data 3. The data to plot Figure 2 and Supplementary Figure 6 is reported in Supplementary Data 11 (the coordinates of the populations are reported in Supplementary Data 2). The data to plot Figure 3B is reported in Supplementary Data 14. Figure 4 combines data from Figure 2 and 3A, the rivers position was obtained from Natural Earth (free vector and raster map data naturalearthdata.com).
The map in Supplementary Figure 1A is made with Natural Earth, the coordinates of the samples showed are reported in Supplementary Data 2. The data to plot Supplementary Figure 1B is reported in Supplementary Data 3. The data to plot Supplementary Figure 2 is reported in Supplementary Data 4. The data to plot Supplementary Figure 3 is reported in Supplementary Data 8. Supplementary Figure 4A and 4B can be plotted using the code in Supplementary Code 1 and 2. The data to plot Supplementary Figure 14 is reported in Supplementary Data 15. The code to plot Figure 3 and Supplementary Figures 8-12 is available on GitHub at the following link: https://github.com/leovallini/ValliniHub2024.

Research involving human participants, their data, or biological material

Policy information about studies with human participants or human data. See also policy information about sex, gender (identity/presentation), and sexual orientation and race, ethnicity and racism.

Reporting on sex and gender	not applicable
Reporting on race, ethnicity, or other socially relevant groupings	not applicable
Population characteristics	not applicable
Recruitment	not applicable
Ethics oversight	not applicable

Note that full information on the approval of the study protocol must also be provided in the manuscript.

Field-specific reporting

Please select the one below that is the best fit for your research. If you are not sure, read the appropriate sections before making your selection.

☒ Life sciences ☐ Behavioural & social sciences ☐ Ecological, evolutionary & environmental sciences

For a reference copy of the document with all sections, see nature.com/documents/nr-reporting-summary-flat.pdf

Life sciences study design

All studies must disclose on these points even when the disclosure is negative.

Sample size	We used all available aDNA data for the period of interest
Data exclusions	We excluded individual genomes with less than 100.000 SNPs genome wide
Replication	No replication was possibile as, due to limited sample size, we used all available aDNA data for the period of interest
Randomization	No randomization was possibile as, due to limited sample size, we used all available aDNA data for the period of interest
Blinding	We applied no treatment on any donor or study subject, so blinding is not applicable

Reporting for specific materials, systems and methods

We require information from authors about some types of materials, experimental systems and methods used in many studies. Here, indicate whether each material, system or method listed is relevant to your study. If you are not sure if a list item applies to your research, read the appropriate section before selecting a response.

Materials & experimental systems

n/a	Involved in the study
☒	☐ Antibodies
☒	☐ Eukaryotic cell lines
☒	☐ Palaeontology and archaeology
☒	☐ Animals and other organisms
☒	☐ Clinical data
☒	☐ Dual use research of concern
☒	☐ Plants

Methods

n/a	Involved in the study
☒	☐ ChIP-seq
☒	☐ Flow cytometry
☒	☐ MRI-based neuroimaging

Plants

Seed stocks	not applicable
Novel plant genotypes	not applicable
Authentication	not applicable
